# MAHILA: a protocol for evaluating a nurse-delivered mHealth intervention for women with HIV and psychosocial risk factors in India

**DOI:** 10.1186/s12913-016-1605-1

**Published:** 2016-08-04

**Authors:** Nancy R. Reynolds, Veena Satyanarayana, Mona Duggal, Meiya Varghese, Lauren Liberti, Pushpendra Singh, Mohini Ranganathan, Sangchoon Jeon, Prabha S. Chandra

**Affiliations:** 1 Division of Acute Care/Health Systems, School of Nursing, Yale University, 400 West Campus Drive, West Haven, CT 06516 USA; 2 Department of Clinical Psychology, National Institute of Mental Health and Neuro Sciences, Bengaluru, 560029 India; 3 Post Graduate Institute of Medical Education and Research, Chandigarh, India; 4 Department of Psychiatry, National Institute of Mental Health and Neuro Sciences, Hosur Road, Bengaluru, 560029 India; 5 Indraprastha Institute of Information Technology (IIIT-D), B-304, Academic Block, Okhla Phase III, New Delhi, 110020 India; 6 Department of Psychiatry, Yale University, School of Medicine, 300 George Street, New Haven, CT 06511 USA

**Keywords:** HIV, Women, Mental health, mHealth, Antiretroviral adherence, LMIC

## Abstract

**Background:**

Women living with HIV are vulnerable to a variety of psychosocial barriers that limit access and adherence to treatment. There is little evidence supporting interventions for improving access and treatment adherence among vulnerable groups of women in low- and middle-income countries. The ***M****obile Phone-Based****A****pproach for****H****ealth****I****mprovement,****L****iteracy and****A****dherence* (MAHILA) trial is assessing the feasibility, acceptability and preliminary efficacy of a novel, theory-guided mobile health intervention delivered by nurses for enhancing self-care and treatment adherence among HIV-infected women in India.

**Methods/Design:**

Women (*n =* 120) with HIV infection who screen positive for depressive symptoms and/or other psychosocial vulnerabilities are randomly assigned in equal numbers to one of two treatment arms: treatment as usual plus the mobile phone intervention (experimental group) or treatment as usual (control group). In addition to treatment as usual, the experimental group receives nurse-delivered self-care counselling via mobile phone at fixed intervals over 16 weeks. Outcome measures are collected at baseline and at 4, 12, 24 and 36 weeks post-baseline. Outcomes include antiretroviral treatment adherence, HIV-1 RNA, depressive symptoms, illness perceptions, internalized stigma and quality of life.

**Discussion:**

The MAHILA trial will provide information about how a mobile health counselling intervention delivered by non specialist nurses may improve access to care and support the adherence and clinical outcomes of women with HIV infection living in low- and middle-income countries such as India.

**Trial registration:**

NCT02319330 (First received: July 30, 2014; Last verified: January 2016)

## Background

There have been dramatic improvements in access to life-saving combination antiretroviral therapy (cART). In 2014, 14.9 million people living with the human immunodeficiency virus (HIV) were receiving cART globally, 13.5 million of whom live in low- and middle-income countries (LMICs) [1]. While cART has markedly improved the prognosis of people living with HIV, deficits in adherence to the spectrum of HIV care pose significant barriers to its long term success [2–4]. Effective cART requires optimal adherence to maintain suppression of viral replication with non-adherence resulting in treatment failure, disease progression and/or the emergence of drug resistance [4–6].

Although India has seen a 19 % decline in new HIV infections, the country has the third highest number of estimated people living with HIV in the world (~2.1 million) [7, 8], of whom, 39 % are women [9]. This amounts to approximately 0.82 million women, given the large population of the country. There is a growing body of evidence indicating that women in India are vulnerable to poor HIV prevention and treatment outcomes [10–18]. Women in India face a variety of inter-related situational and psychosocial barriers that may limit their access to HIV care and adherence to cART over time including low literacy, stigma, discrimination and low social support [19–21]. Depression, a prominent predictor of poor adherence to cART [22–26], is also prevalent among women living with HIV in India in whom it is largely under diagnosed and undertreated [27, 28].

Good adherence is critical in HIV prevention and treatment success, yet little attention has been given to the development of interventions focused on women living with HIV and depression or other psychosocial risk factors for nonadherence. Interventions that improve adherence and reduce the barriers that women face in adhering to cART are urgently needed. Our preliminary work indicates that a theory-guided phone intervention originated in the U.S. is well suited to the Indian context given the widespread use of mobile phone technology [29]. This multi-dimensional, patient-centered approach builds patient-provider rapport, establishes sources of support, and enables and empowers problem solving to address situational and psychosocial barriers to care [29]. Other HIV adherence interventions using mobile health (mHealth) approaches in LMIC’s have mainly relied on text-based reminders [30–33]. Women with low literacy skills may find text message based interventions challenging. Further, these messages serve mainly as memory prompts to take the medications and do not address psychosocial factors that influence adherence behaviour [32].

Following initial formative work to refine the mobile phone-based adherence intervention for delivery in India, we trained non specialist nurses to deliver the intervention. We are now evaluating whether the low cost, mHealth counselling intervention is an effective, feasible and acceptable way to improve the treatment outcomes of women in India who are affected by HIV with and inter-related mental health and psychosocial risk factors. Here we describe the protocol of the ongoing randomized, single blind (rater), controlled trial. The SPIRIT guidelines were adhered to for the reporting of this manuscript.

## Methods

### Study aim and design

**Fig. 1:**
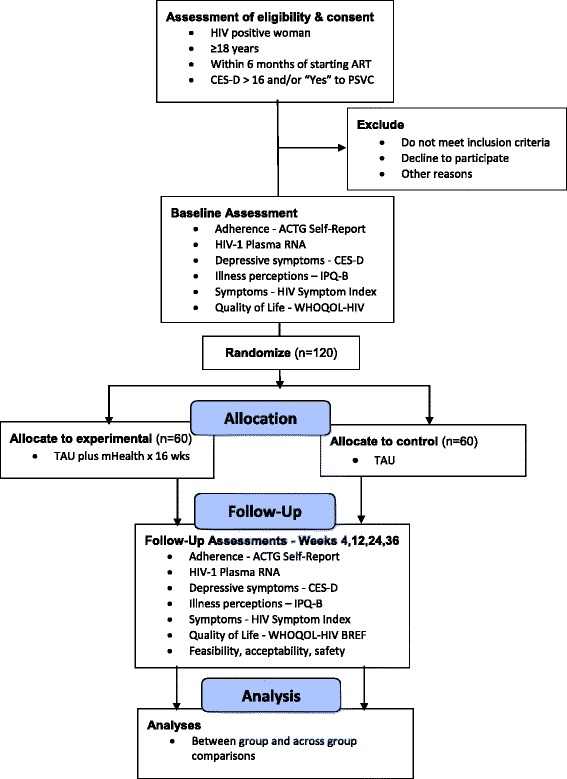
MAHILA flowchart

The MAHILA project is being conducted through a Collaborative Research Partnership between investigators at the National Institute of Mental Health and Neuro Sciences (NIMHANS) and Yale University. The study is designed to evaluate the feasibility, acceptability, fidelity and preliminary efficacy of a standardized mobile phone intervention in a randomized, controlled pilot study in which 120 HIV-infected women are randomized to treatment as usual (TAU) alone or TAU plus the mHealth (delivered over 16 weeks). After baseline assessment, women are randomly assigned to treatment condition in a 1:1 allocation and outcomes (adherence, HIV-1 RNA plasma [viral load], mental health) are evaluated at 4, 12, 24 and 36 weeks post-randomization (see Fig. 1). The primary hypothesis is that TAU plus the mobile phone intervention will be more effective than TAU in improving adherence to cART and clinical outcomes at 24 weeks post-randomization. Other hypotheses are that the mobile phone intervention will be feasible and acceptable to women living with HIV, fidelity of the intervention will be maintained, and the intervention will improve HIV adherence by concurrently addressing prominent barriers (e.g., depressive symptoms) and improving knowledge, support and problem solving for better engagement in care and adherence.

### Study setting

The ***M****obile Phone-Based****A****pproach for****H****ealth****I****mprovement,****L****iteracy and****A****dherence* (MAHILA) project is conducted at the government-sponsored HIV treatment clinic (ART Centre) of the Belgaum Medical College Hospital, in the state of Belgaum, Karnataka and at NIMHANS, Bengaluru, South India. Recruitment of participants and data collection is conducted by trained project staff at the ART Centre in Belgaum. The mobile phone intervention is delivered by trained non specialist nurses at NIMHANS. Delivery of the intervention from a central site allows for prevention of within site dispersion of intervention content to the control group and it enables close monitoring of the fidelity of intervention delivery by the project investigators with mental health expertise at NIMHANS. These non specialist nurses are trained as part of this study on the clinical and psychosocial aspects of HIV/AIDS, basics of mental health screening, phone based counseling and the self-care intervention in four separate workshops.

Belgaum is situated in North-West Karnataka and shares its borders with two neighbouring states – Maharashtra and Goa. It is the largest district in the state with a population of 4.8 million [34]. The district has the highest overall HIV prevalence (1.43 %) in the state of Karnataka with prevalence greater in rural than urban areas [35].

High rates of migration and a tradition called the *Devdasi* system is prevalent in several parts of the district [36]. Devadasi is a religious practice whereby parents “marry a daughter to a deity or a temple”. While the Devadasi practice has a long tradition in parts of India, many devadasis today become religiously sanctioned prostitutes [37–39].

### Sample size and eligibility criteria

One hundred and twenty participants will be enrolled, with 60 in each treatment arm. This sample size is consistent with developmental research with the primary aim of assessing the feasibility, acceptability and preliminary efficacy of a new behavioral intervention [40]. It is also large enough to allow for a diverse set of participant characteristics representative of the target population.

To be eligible, a woman must be 1) able to give informed consent, 2) able and willing to be contacted by mobile phone, 3) 18 years of age or older, 4) speak Kannada, English or Hindi, 5) HIV+ and taking cART for less than six months, and 6) screen positive for depressive symptoms or psychosocial risk factors. Women are screened for depression with the Centre for Epidemiologic Studies Depression Scale (CES-D) [41] and for psychosocial risk factors with the Psychosocial Vulnerability Checklist (PSVC). The PSVC was developed by the researchers based on a review of studies among women with HIV, especially in LMICs [26, 28, 42, 43]. It consists of 14 items which assess level of financial strain, social support, violence, substance abuse, mental health problems and suicide ideation.

Women are excluded from participation if they have any condition that, in the opinion of the site investigator, would compromise their ability to participate. Women who screen positive for active suicidal ideation are also excluded. All women with > 16 CES-D or active suicidal thoughts are referred for treatment per a standardized, written protocol.

### Recruitment, enrollment and randomization

Potential participants are invited to participate by a study team member in the ART Centre in Belgaum, who then explains the study, obtains informed consent and screens them for eligibility. Eligible participants then complete the baseline assessments.

Each participant is provided with a basic mobile phone. This mobile phone is used for proactive delivery of the intervention to participants in the TAU + mobile phone arm. To enhance methodological rigor, mobile phones are also distributed to the participants in the TAU arm to avoid a placebo effect and for ethical reasons: for instance, the phone may be used by participants in either arm to call the study nurse ad hoc for health-related advice or assistance. It is also used by study staff for purposes of establishing contact or collecting data if necessary. The mobile phones are provided with Subscriber Identity Module (SIM) cards under a Closed User Group (CUG) connection, in which the participant can make calls to the CUG number of the study nurse and can receive calls from the same number. The subscription is paid for by the project on a monthly basis. This approach ensures that the phone is not used for personal calls and it overcomes the difficulty of providing regular “top ups” of phone currency.

Participants are randomized to a treatment condition following the baseline assessment in a 1:1 allocation ratio to one of the two treatment arms. Computer-generated, block randomization is used to ensure balanced representation in the two treatment arms. Sequentially numbered opaque sealed envelopes serve as the allocation concealment method. The Belgaum study staff are blinded from the randomization assignment. Once a new participant is enrolled, the Belgaum staff notify the project coordinator who is located in the project research office at NIMHANS. The project coordinator initiates the randomization procedures and hand delivers notification of randomization assignment to the study nurses who then make contact with the participant by mobile phone and inform them of the treatment assignment.

### Study interventions

#### Control group – TAU

All participants receive TAU as prescribed by the Indian National ART guidelines [44]. In addition, referral to psychiatric care is made by project staff for depressive symptoms or suicidal thoughts at baseline and follow-up assessments. Further, as noted above, all participants may initiate ad hoc contact with a study nurse to confer about health-related matters. The time, date, and content of any calls initiated by the patient are documented by the nurse on a project form for analysis. Time matched attention in both arms is not possible given the individualized approach with variable frequency and length of the intervention calls for each participant.

#### Experimental group – TAU plus nurse-delivered mobile phone intervention

The mobile phone intervention is delivered proactively by nurses via mobile phones at a convenient time identified by the participant for up to 16 weeks. Calls are made by the nurse at baseline and at least two times per week during weeks 1–4, once a week during weeks 5 to 10, and then at weeks 14 and 16. More frequent calls can be made at the discretion of the nurse or if initiated by the participant as in the TAU arm. Up to 6 attempts are made by the nurse for each scheduled contact. As noted above, participants also have the option of initiating calls to confer with the nurse on health-related matters between scheduled calls (date, length and content of each call are recorded by the nurse). A script is used to guide delivery of the critical elements of the manualized mobile phone intervention, but the duration and content is individualized to each participant.

The multi-component mobile phone intervention was developed through a series of studies guided by application of the Leventhal self-regulatory model of illness behavior to HIV [29]. It has been tested in the U.S. and adapted for delivery to women in India during Phase 1 of project MAHILA. At the core of the approach is a trained nurse who contacts patients proactively by mobile phone at regular intervals. A structured, patient-centered, counseling approach is used to engage and develop the individual’s capacity for productive self-care behavior. Self-care behavior of the individual living with a chronic illness is essential to the effectiveness of medical treatments since most of the day-to-day management takes place outside of health care settings. A host of research conducted by Leventhal and others has shown that the way in which individuals interpret or make sense of their illness (illness representation) drives the selection of coping/self-care behaviors (e.g., adherence to medication) [45–51]. Illness representations are highly individual, influenced by a host of internal and external information that are often ambiguous, fluctuate daily, and are affected by situational variation. They may or may not be compatible with medical norms and influence how new health information is processed and acted upon; any health information provided will interact and be accepted or rejected based on the patient’s pre-existing illness representations. Thus, a central element of this mobile phone intervention is assessing how the patient comprehends her illness in the context of her life circumstances and past experiences. Areas of risk and strengths are identified, corresponding health information and support is provided and skills developed that are individualized to the patient’s schema and situational context. The premise is that in so doing the content is viewed as more meaningful and is more readily integrated into the individual’s cognitive schema and acted upon in problem solving efforts to manage barriers and emotional responses that surround both the primary difficult experience of living with an illness condition in the context of the individual’s situational challenges. Further, the phone contact provides a means of facilitating coordination of services, continuity of care, and patient monitoring.

The overarching objective is to ensure that the patient in the TAU + mobile phone arm is provided with accurate information and support, individualized to patient’s knowledge level and situational context. This is intended to stimulate development of a perceptual schema and skills to engage resources and problem solve to manage common barriers and emotional responses that surround both the primary difficult experience of living with HIV and taking antiretroviral therapy in the context of situational demands and challenges (e.g., stigma/disclosure, competing demands/priorities (e.g., family/child care), side effects, depressive symptoms, social support). A combination of strategies are used. The five-step process begins with an assessment of the patient’s representation of HIV and medication, areas of risk are identified, and corresponding, key health information and affective support are provided in accordance with the participant’s priorities and contextualized to the patient’s schema and situational context to build knowledge and skills to problem solve to sustain adherence/self-care behavior as indicated. Appropriate referrals are generated as indicated.

The key components of the intervention include: (1) providing the patient with an individualized program that is congruent with patients’ social and cultural context; (2) integrating screening for depression and other concurrent risk factors; (3) enabling proactive problem solving to aid participants in overcoming factors that may impede their engagement in treatment; (4) improving early recognition of barriers and referrals; and, (5) providing a mediator (the study nurse) between the health system and the participants. For example, the study nurse will screen for the presence or worsening of depression and facilitate the engagement of the participants in appropriate mental health care by: (a) providing information contextualized to the cognitive representation and concerns (e.g., stigma/disclosure) of the patient; (b) scheduling of appointments; (c) follow-up. This provides an opportunity for continuity and coaching through threats to treatment adherence that may manifest over time. The sessions are interactive and patient-centered. The nurse listens actively and uses theory-directed open-ended questions and probes. Communication is positive, non-judgmental and encouraging (See summary Table 1).

**Table 1:** Summary of key elements of the mobile phone intervention

Key elements	
1.	Proactive, nurse-delivered calls by mobile phone at time convenient to the participant;
2.	Call sessions are interactive and patient-centered. The nurse listens actively and uses theory-directed open-ended questions and probes. Communication is positive, non-judgmental and encouraging.
3.	Content of calls is individualized to the participant’s cognitive representations, concerns (e.g., stigma/disclosure) and sociocultural context;
4.	Screening for depression and other concurrent psychosocial risk factors;
5.	Early recognition of barriers and referrals - Coaching through threats to care that may manifest over time;
6.	Building of problem solving skills to aid participants in overcoming factors that may impede their engagement in treatment;
7.	The nurse plays a mediating role between the health system and the participant. Enhanced continuity of care.

#### Fidelity

To ensure treatment fidelity, the nurses are provided with Android smart phones so that the intervention sessions can be recorded using an application. With the consent of participants, the Android application is enabled to record any incoming or outgoing calls made on the phone which are saved on a secure laptop that is password protected. The recorded calls are checked randomly by the study principal investigators and the study nurses are provided with feedback and guidance on the content and delivery of counseling sessions as needed.

### Measures

All study assessments (baseline and 4, 12, 24 and 36 weeks post-randomization) are conducted by trained study staff who are blind to treatment allocation. Assessments are typically conducted in person in the Belgaum ART Centre, but there is some allowance for mobile phone-based follow-up assessments in cases where participants are unable to return to the study site. The data from the forms can be exported in different formats, such as Microsoft Excel file format (.xls) for further processing.

*Adherence* is measured with the AIDS Clinical Trails Group (ACTG) Adherence Questionnaire [52]. The questionnaire queries the patient on the number of doses missed of an ART medication during each of the 4 days before a clinic visit (e.g., “How many doses did you miss yesterday, the day before yesterday, 3 days ago, and 4 days ago?”). It has been used widely in the U.S. and India and has shown good validity and reliability [53].

*Plasma HIV-1 RNA* concentration is used as the clinical criterion and primary outcome variable. Plasma HIV RNA concentration is measured in copies per millilitre (Roche Amplicor HIV-1 Monitor Test). The threshold for detectability of HIV viral load 50 copies/mL.

*Depression* is measured with the Centre for Epidemiologic Studies Depression Scale (CES-D) [41, 54]. CES-D (α = 0.88-0.91) is a 20 item screening tool for depressive symptomatology. All items are rated using a 4 point ordinal scale with the responses based on occurrence of symptoms in the past one week.

*Illness representation* is measured with the Brief Illness Perception Questionnaire (IPQ-B), a 9-item questionnaire designed to rapidly assess cognitive and emotional representations of illness [55]. The IPQ-B uses a single-item scale approach to assess perception on a 0–10 response scale, with higher scores representing more threatening illness perceptions. The scale has demonstrated good test-retest reliability (Pearson correlations 0.24-0.73) and moderate to good correlations when tested for concurrent validity (Pearson correlations 0.32–0.63).

*Symptom Distress* is measured with the HIV Symptom Index, a 20-item valid and reliable (α = 0.92) measure of overall HIV symptom frequency and level of bothersomeness [56].

*Stigma* is measured with a 10-item measure of internalized stigma. The scale was adapted from the Stigma Scale which was developed in South India and measures enacted, felt normative and internalised stigma [57]. Internalised stigma experienced by respondents is measured on a 4 point scale running from 0 (not at all) to 3 (a great deal).

*Quality of life* is measured with the World Health Organization (WHO) Quality of Life (QOL) HIV short version (WHOQOL-HIV BREF), a multidimensional 31 item instrument used to assess quality of life of persons with HIV [58]. WHOQOL-HIV BREF has been used and validated in the Indian HIV population [59–61].

*Feasibility, acceptability, and fidelity* of the intervention and study protocol are measured with protocol specific tools. The assessment includes: 1) The ratio of eligible study participants to those enrolled; 2) Number of scheduled study visits completed at 4, 12, 24, and 36 weeks; 3) Attrition between baseline and follow-up; 4) Reason for premature drop-out; 5) Number of phone calls that were made on schedule; 6) Level of participation in intervention sessions including the total number sessions, number of sessions completed without break offs, number of break offs, length (minutes) of sessions; 7) Congruence of topic/content discussed on calls with protocol; 8) Patient and study nurse satisfaction with intervention content, mode of delivery, and protocol; and, 9) Adverse events.

A study-specific intervention assessment tool is used to evaluate content fidelity and quality of the interaction and a log is maintained that details technical challenges faced by the team (e.g., problems related to mobile phone, SIM card).

### Data storage and monitoring

A web-accessible and interactive database system is used for the storage and exploration of the study data. The data collection framework uses an open-source relational database management system, MySql, at the back-end and Python-Django at the front end with a MVC (Model-View-Controller) architecture. The MVC architecture makes it easy to develop new functionalities and extend the capabilities of the framework. In the front-end we have created forms for collecting the baseline and follow-up data. The use of a data-collection framework offers many advantages over conventional paper-based forms. For example, sharing data across partners is easier and the forms have a feature of preliminary validation, i.e., a user must enter values which are in the valid range otherwise the form refuses to accept the input.

All study participants are assigned unique study identifiers that appear on all data collection instruments, tapes, documents, and files. Data collection instruments and the study database contain no client identifying information or record of HIV status and are stored in a secure, double-locked cabinet. Personal information needed for tracking and informed consent are stored separately from other data in double-locked, fireproof, water resistant safe in the ART Centre. No identifying information will leave the clinic or be entered into the project database.

Data are entered into study-specific laptops. Microsoft Access is used for storage and management. The master study dataset is stored on a dedicated Windows file server that can only be accessed by authorized study staff. All data are password protected.

A Data Safety and Monitoring Board (DSMB) has been constituted to review the progress and safety of study procedures and address unanticipated problems involving risks to subjects or other serious adverse events. The DSMB meets every three months.

### Statistical analysis

Data will be described using univariate analysis (distributions, frequencies, and means). The intervention assignment will be examined in relation to HIV-1 plasma and cART adherence. Analyses will be intention-to-treat. As this is a small sample size we will only be able to evaluate parsimonious models to explore effect of the intervention, and not include all mediating and potentially confounding variables. Generalized estimating equations (GEEs) will be used to model adherence rates to account for the multiple measurements of each subject and allow all subjects, regardless of the number of visits, to be included in the analysis. Analyses also will be conducted within-person, comparing baseline to follow-up session responses. As GEEs require strong assumptions regarding missing data (missing completely at random), analyses will be repeated using the subset of subjects with complete data.

## Discussion

To our knowledge, the current study is the first clinical trial to examine the effects of a nurse delivered mobile phone based self-care intervention for women with HIV infection who have depression or psychosocial risk factors in a LMIC [32]. Results from this study will inform the development of interventions for achieving improved clinical benefits and recommendations for mHealth service delivery, from the clinic to national and international policy levels. Findings will also contribute to understanding how mobile technology interventions motivate better health and self-care among women with HIV. Furthermore, in addition to outcome data, respondent and provider satisfaction that are collected as part of this study protocol may provide guidance to HIV and mHealth experts who are seeking to evaluate mobile phone programs for HIV prevention, care, and treatment.

The protocol focuses on mHealth impact on psychological behavioral and physical health; assesses implementation issues unique to mHealth interventions; and also looks at the gender intentionality of the intervention, all of which are priorities for evidence generation in the area of mobile phones for health among women in LMICs. The study will also help us in identifying any risks that women might face related to the mobile intervention such as disclosure issues, any increase in stigma or escalation of partner violence.

There are a few study limitations worth noting. Self-reported adherence to cART has notable limitations [62–65]. We have, however, measured HIV-1 RNA in plasma which is the gold standard for monitoring the efficacy of HIV treatment and measuring the validity of adherence reports. If a patient’s virus is known to be sensitive to current therapy, a detectable viral load likely represents poor adherence [66]. The health behavior constructs we are assessing have not often been evaluated in mobile phone interventions, but prior research and focus group data from other projects suggests that the proposed constructs are affected by mHealth programs [29, 67, 68]. In addition, we will not be able to control external factors that may confound study results, such as medication supplies or health system challenges, weather, civil or political influences, or other health programs occurring in study communities during the intervention period, although we will document these contextual factors to help with interpretation of findings.

The study has several strengths. It is the first trial using a mHealth non text based intervention among women with HIV infection in a LMIC; biases and contamination have been minimised by adequate allocation concealment and by ensuring that the teams of assessors and interventionists are in two different geographical locations. The intervention itself is theory driven and is based on the self-regulatory model of ART adherence [29]. Both groups of women have been given mobile phones to ensure blinding and remove any effect of owning a phone rather than receiving an intervention. Adequate training and supervision is provided to the nurses including a periodic review of call recordings by the investigators to ensure fidelity. Standard operating procedures have been developed for women who report mental health problems, suicidality or domestic violence. Any adverse effects or difficulties related to the intervention will be recorded. Multiple outcomes including adherence, mental health outcomes, quality of life and biological parameters of disease progression (e.g., HIV-1 RNA) are measured. An important outcome that has not been studied in previous trial will include respondent and provider satisfaction with the intervention.

## Conclusions

This nurse led mobile phone intervention for improved self-care among women with HIV infection who have depression and/or psychosocial vulnerabilities will provide important evidence for the usefulness, feasibility and acceptability of this novel intervention. Results of this trial will be important in informing future mHealth interventions for this population and for women with other chronic medical illness in LMICs.

## Abbreviations

ACTG, AIDS clinical trials group; ART centre (antiretroviral centre) - a government-sponsored HIV treatment clinic; cART, combination antiretroviral therapy; CES-D, centre for epidemiologic studies depression scale; CUG, closed user group;.xls - microsoft excel file format; DSMB, data safety and monitoring board; GEEs, generalized estimating equations; HIV, human imuunodeficiency virus; HIV-1 RNA plasma (viral load) - a quantitative measurement of viremia; ICMR, Indian council of medical research; IPQ-B, brief illness perception questionnaire; IRB/ECs, institutional review board or ethics committees; LMICs, low- and middle-income countries; mHealth, mobile health, a term used for the practice of medicine and other health care services supported by mobile devices; MVC (Model-View-Controller), a software architectural pattern mostly (but not exclusively) for implementing user interfaces; MySql, an open-source relational database management system; NIMHANS, national institute of mental health and neuro sciences; PSVC, psychosocial vulnerability checklist; SIM card, subscriber identity module card is a portable memory chip used in cell phones; TAU, treatment as usual; WHOQOL-HIV BREF, world health organization quality of life HIV short measure
